# Kaleidoscopic Chloroma: An Unusual Case of Myeloid Sarcoma

**DOI:** 10.7759/cureus.75244

**Published:** 2024-12-06

**Authors:** Anil Prasad, Binod Kumar, Sanghamitra Jena, Minakshi Mishra, Nilanjan Sarkar

**Affiliations:** 1 Pathology, Tata Main Hospital, Jamshedpur, IND; 2 Dermatology, Tata Main Hospital, Jamshedpur, IND; 3 Surgical Oncology, Tata Main Hospital, Jamshedpur, IND; 4 Radiology, Tata Main Hospital, Jamshedpur, IND

**Keywords:** acute myeloid leukemia (aml), chloroma, intestinal obstruction, myeloid sarcoma, myeloperoxidase

## Abstract

We report a 37-year-old male patient who had nonbilious vomiting, no passage of flatus, and recurring abdominal pain. This patient had de novo intestinal myeloid sarcoma (MS), a rare and chameleonic presentation of acute leukemia of myeloid origin. The initial diagnostic evaluation suggested Koch's abdomen, and surgical excision of the bowel was performed with a clinical suspicion of Koch's or lymphoma. However, following extensive evaluation, it was diagnosed as chloroma. The rarity of intestinal MS without a pre-existing hematolymphoid malignancy presents a substantial diagnostic challenge, as few cases are documented in the literature.

## Introduction

Myeloid sarcoma (MS) is defined by the World Health Organization (WHO) as “a tumor mass consisting of myeloblasts with or without maturity that occurs outside the bone marrow" [1]. Due to the rarity of this disorder, most of the relevant literature comprises case reports. This report aims to add to the current literature body and highlight the issue of misdiagnosis or delayed diagnosis in these patient subsets. Among all acute myeloid leukemia (AML) subtypes, MS is still classified as having a unique clinical presentation. MS can appear as an isolated tumor or be associated with myeloproliferative disorders (MDS), myelodysplastic neoplasms (MPN), AML, or a combination of MDS and MPN. Just one to two percent of all myeloid leukemia cases presented as MS. The incidence of gastrointestinal MS is low, around 6.5% [2]. With a frequency of two cases per million individuals, isolated MS is an uncommon condition.

The clinical manifestation of MS differs depending on the location and size of the lesions. As a result, there is little clinical understanding of the disorder, its presentation, and its novel targeted therapy. A large portion of the literature is limited to case reports and occasional retrospective series. Even though MS is rare, it seems to be becoming more common; up to 9% of people with AML or chronic myeloid leukemia (CML) have it. This increase is partially due to allogeneic hematopoietic stem cell transplantation or treatment with novel targeted agents, such as small molecule inhibitors, nucleoside analogs, histone deacetylase inhibitors, and DNA methyltransferase inhibitors, which have been shown to increase the risk of extramedullary relapse in AML. The current study may contribute to a better understanding of de novo MS.

## Case presentation

**Figure 1:**
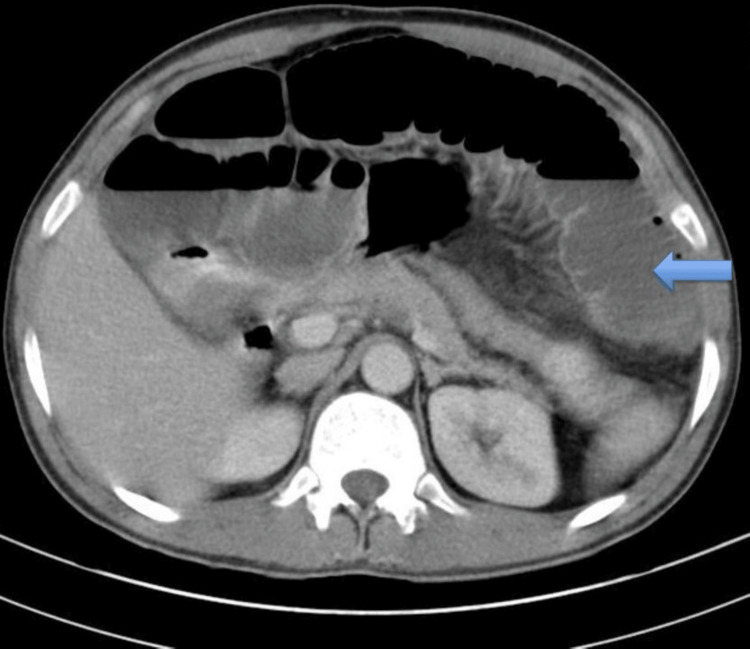
CECT axial view of the abdomen with the blue arrow, demonstrating small intestinal obstruction CECT: contrast-enhanced computed tomography

A 37-year-old male, a milkman by profession, was admitted to the hospital with nausea, non-bilious vomiting, recurrent abdominal pain, and chronic constipation for one month. His past history was not insignificant. A physical examination revealed that he had discomfort and tenderness in the right lower abdomen. Biochemistry and hematological examinations have not shown any significant findings. Clinical differential is suspected like Koch’s or small bowel carcinoma. Abdominal imaging was suggestive of intestinal obstruction as multiple air-fluid levels were seen. After surgical consultations and preliminary diagnosis of acute abdomen, the patient was investigated for ultrasonography abdomen followed by computed tomography (CT) (Figure 1) examination, which was suggestive of multiple dilated small bowel segments along with air-fluid level and focal obstructive lesion, possibly intussusception with associated small bowel obstruction. The patient underwent exploratory laparotomy, ileal resection, and double barrel ileostomy. Koch's, or small bowel carcinoma, was suspected as a differential diagnosis based on radiological findings.

**Figure 2:**
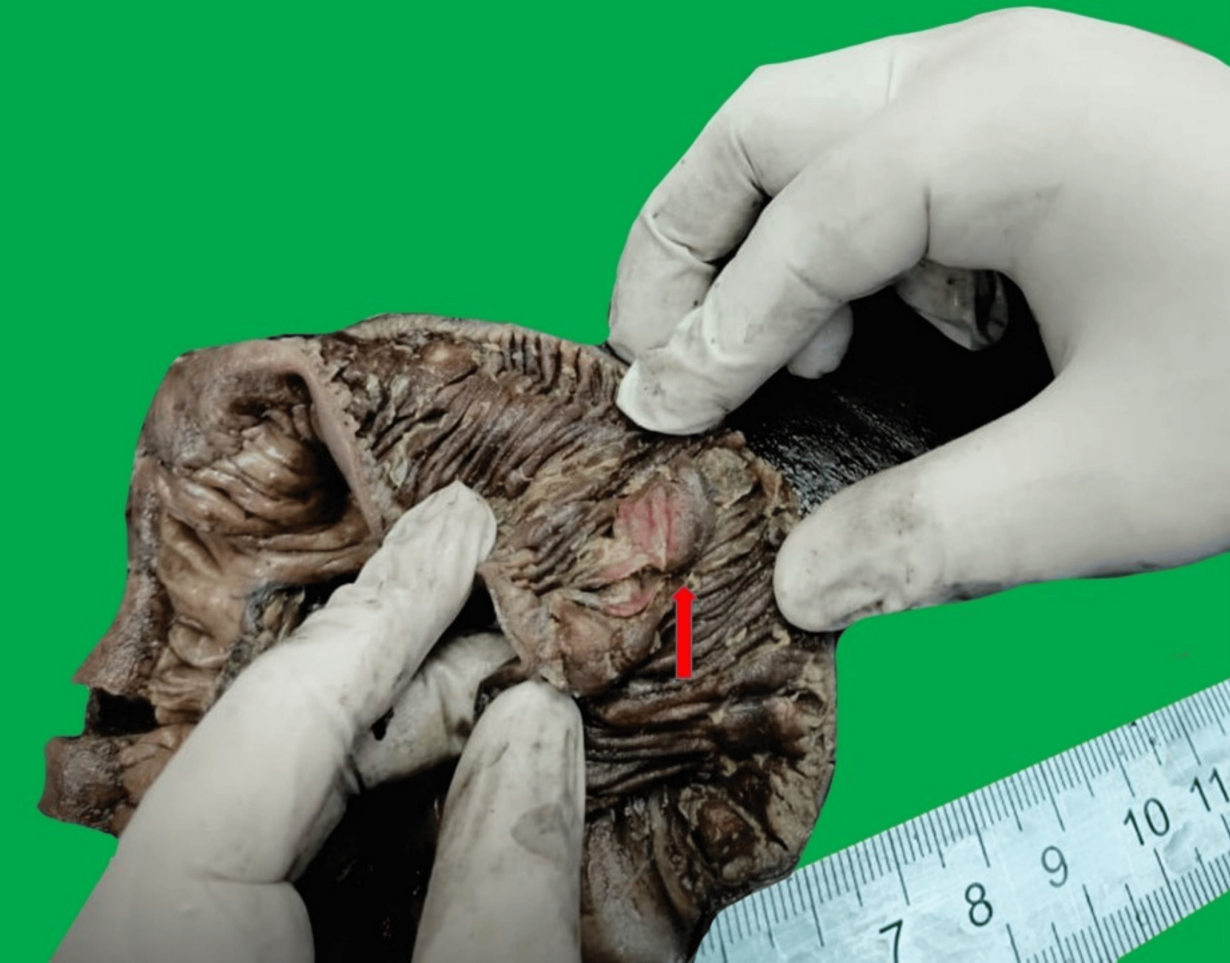
Resected small bowel specimen, cut section, showing a polypoidal lesion (pink arrow)

On histopathology, gross and cut section examinations of the small intestine reveal irregular, numerous polypoidal masses that have infiltrated the serosal surfaces and occupied the intestinal lumen (Figure 2).

**Figure 3:**
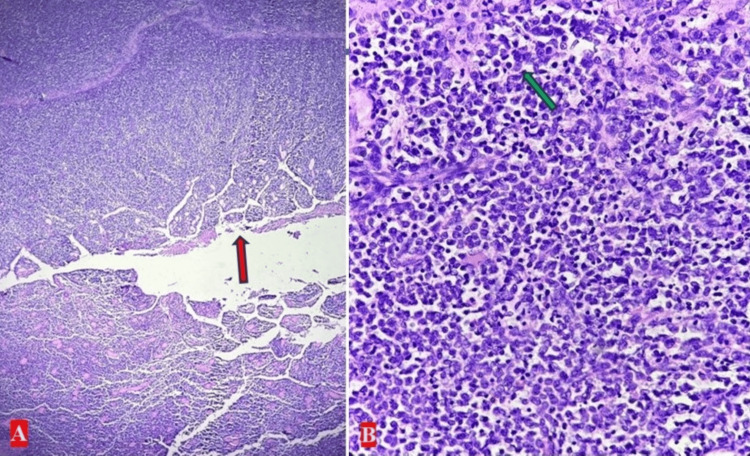
(A) Scanner view photomicrograph demonstrating intestinal mucosal architecture effaced by infiltrating myeloblasts (pink arrow) (X40, hematoxylin and eosin). (B) Photomicrograph at X400 magnification demonstrating sheets of immature myeloid cells with large, hyperchromatic nuclei and fine chromatin (green arrow)

Microscopic examination shows loss of normal bowel architecture, diffuse proliferation of non-cohesive cells with nuclear enlargement, nuclear atypia, hyperchromasia, and scant to moderate cytoplasm. Frequent mitotic figures and apoptosis were observed (Figure 3).

**Figure 4:**
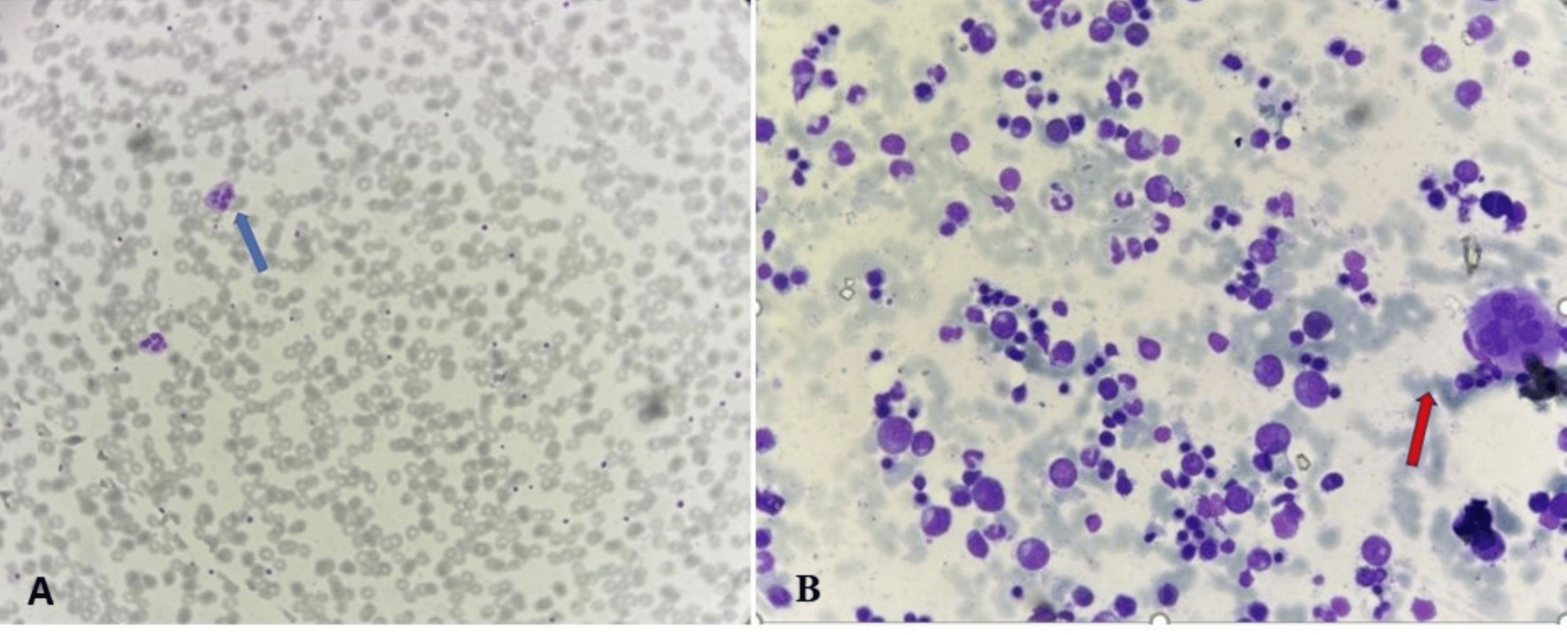
(A) Peripheral smear Leishman staining at X100 magnification showing a normal blood picture, with neutrophils (blue arrow). (B) Bone marrow aspiration Leishman staining at X400 magnification showing normal bone marrow, with a megakaryocyte (pink arrow)

Immunohistochemical analysis showed these cells were positive for MPO, CD43, CD68, and CD117 with a high Ki index of 70%. CD34 was negative. MS was diagnosed in the patient based on these findings. Bone marrow aspiration and biopsy examination did not demonstrate any involvement of myeloid neoplasm in the marrow (Figure 4). The patient was diagnosed with isolated intestinal MS.

## Discussion

The first description of chloroma was made by Dr. A. Barns, a British physician, in 1811 [3]. Chloromas can occur in relapse cases of AML that have been previously treated and are in remission [4]. "Chloroma" originates from the Greek "chloros," meaning green, highlighting the distinctive greenish hue these tumors may display. This coloration results from high levels of myeloperoxidase in immature myeloid cells [5]. Dock and Wardthin's 1902 study was the first to establish the correlation between chloroma and acute leukemia. Most small bowel tumors are metastases (50%). The primary malignant lesions that occur most are carcinoids (44.3%), adenocarcinomas (32.6%), and lymphomas (14.7%). The incidence of GISTs (7.2%) and sarcomas (1.2%) is low [6]. MS has four clinical presentations: 1) with AML; 2) as a precursor to AML; 3) associated with MDS progressing to leukemia; and 4) as isolated cases. Pathologically, it is categorized into blast cell, partially differentiated, and differentiated types based on the proportion of immature myeloid cells [7]. Blastic variants of MS present as myeloblasts with few differentiated promyelocytes. Routine histopathological examination often misses the diagnosis of MS, particularly in the following situations: when it is located in uncommon sites like the small intestine, as a solitary lesion with no leukemia detected in peripheral or bone marrow smears, when it lacks a greenish hue, and when its cells are poorly differentiated [8]. Lymphoma can be dangerously mimicked by intestinal MS. This can result in poor outcomes and improper therapy if misdiagnosed. The preferred sites of metastasis are skin, bones, and lymph nodes.

Imaging studies like CT scans of small bowel and colon MS show diverse findings, including mass lesions with luminal narrowing, polypoidal appearance, thickened bowel wall, or a combination of these features [9]. Accurate diagnosis requires a broad panel of immunohistochemical stains to avoid misdiagnosis. In cases without bone marrow involvement, fluorescent in situ hybridization studies and mutation analysis should be performed on formalin-fixed paraffin-embedded (FFPE) samples. The differential diagnosis of MS is broad. Systemic chemotherapy, particularly AML-type induction chemotherapy, may delay AML transformation and prolong survival [10]. Treatment guidelines are lacking, varying based on location, initial versus relapse diagnosis, patient age, and performance status. Current treatment involves conventional AML chemotherapy with or without radiotherapy. Surgery may be considered for debulking, symptomatic relief, or diagnostic confirmation before systemic treatment. Hematopoietic stem cell transplantation is an option for relapsed/refractory patients or as consolidation therapy [11]. The prognosis for MS is poor, with most patients succumbing to the disease quickly. Few achieve long-term remission. Untreated MS typically transforms into AML within 10 months, though longer survival (>16 years) has been reported in rare instances [12].

For a precise diagnosis of MS, appropriate imaging is required to locate the lesion site, aid in assessing complications, and guide biopsy. Subsequently, meticulous histological examination and immunohistochemistry are needed.

## Conclusions

MS is a rare, aggressive cancer arising from myeloid cells that can appear anywhere in the body. Its varied presentation makes diagnosis difficult, requiring imaging, biopsy, immunohistochemistry, and genetic testing. Treatment options, including chemotherapy, radiation, surgery, and potentially stem cell transplantation, are limited and depend on the individual case. The prognosis is poor, with a high risk of developing AML. More research, particularly large-scale clinical trials, is needed to improve diagnosis, treatment, and explore targeted therapies.
